# Robotic splenectomy with ex vivo bench surgery and hemi-spleen autotransplant: the first report

**DOI:** 10.1007/s11701-016-0635-3

**Published:** 2016-08-11

**Authors:** Pier Cristoforo Giulianotti, Despoina Daskalaki, Luis F. Gonzalez-Ciccarelli, Francesco M. Bianco

**Affiliations:** Division of General, Minimally Invasive and Robotic Surgery, Department of Surgery, University of Illinois Hospital and Health Sciences System, 840 S Wood St, Chicago, IL 60612 USA

**Keywords:** Robotic splenectomy, Ex vivo surgery, Partial splenectomy, Hemi-spleen, Autotransplant, Case report

## Abstract

**Background:**

We describe our experience with what is, to our knowledge, the first case of robotic assisted ex vivo partial splenectomy with auto-transplantation for a benign non parasitic cyst.

**Materials and Methods:**

The patient is a 32 year-old female with a giant, benign splenic cyst causing persistent abdominal pain. Preoperative imaging showed a cystic lesion measuring 8.3 × 7.6 cm, in the middle portion of the spleen. Due to the central location of the bulky lesion a partial splenectomy was not feasible. As an alternative to a total splenectomy, a possible reimplantation of hemi-spleen after bench surgery was offered. We proceeded with a robotic total splenectomy and bench hemisplenectomy, preserving the lower pole and a portion of the middle segment of the organ. A robotic reconstruction of the splenic vessels was then performed intra-abdominally. The reperfusion was optimal.

**Results:**

The total operative time was 305 min, with 78 min of robotic time. Postoperative ultrasound confirmed a patent arterial and venous flow. The postoperative course was uneventful and the patient was discharged on postoperative day 4. The pathology report was consistent with epithelial cyst of the spleen. The patient is doing well at 6-month follow-up.

**Conclusions:**

The optimized vision and dexterity provided by the robotic system allowed a safe and precise reconstruction of the splenic vessels, even in a deep and narrow operative field. Partial splenectomy with autotransplantation of the organ was thus achieved, avoiding a total splenectomy in a young patient.

## Introduction

The treatment of benign splenic lesions has evolved over time. While total open splenectomy for non-parasitic splenic cysts is still considered a surgical option, the risk of overwhelming post splenectomy infections (OPSI), especially in younger patients, has prompted the development of newer, less invasive approaches [1]. The best described techniques are totally laparoscopic or robot-assisted partial splenectomy, but they are still considered technically challenging or impossible for very large cysts or cysts involving the splenic hilum [1–3]. Hence, more options are beginning to evolve, including open ex vivo partial splenectomy and auto-transplantation [2]. We describe here our experience with what is, to our knowledge, the first case of robotic assisted ex vivo partial splenectomy with auto-transplantation for a benign non parasitic cyst.

## Materials and methods

**Fig. 1:**
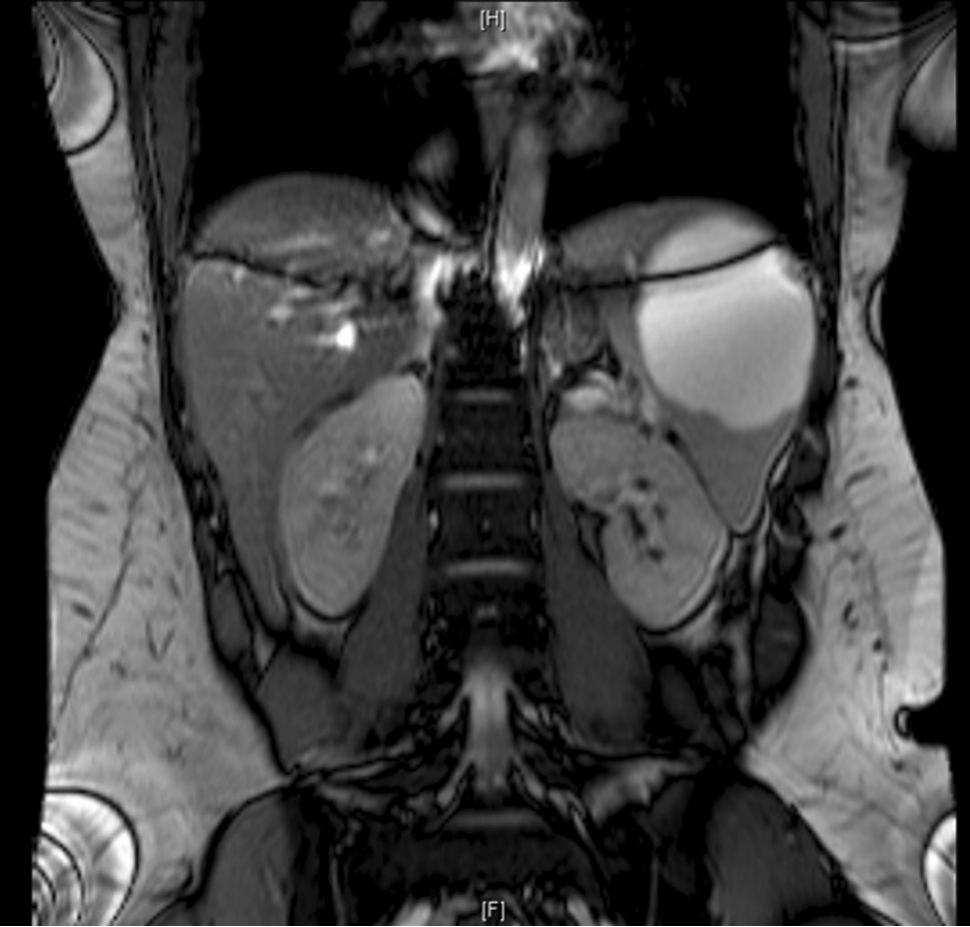
Pre-operative MRA revealing a large splenic cyst

We present the case of a 32 year old female, body mass index (BMI) of 30 kg/m^2^, with a history of endometriosis and appendectomy who presented for persistent abdominal pain, requiring opioids for relief. The pain was referred as dull and achy in nature, concentrated in the upper abdomen and had no link to diet or bowel habits. She had no other complaint. Physical examination did not reveal any specific findings. CT-scan of the abdomen was performed and revealed an 8.3 × 7.6 cm splenic cyst with normal splenic vascularization. Abdominal MRA confirmed these findings (Fig. 1).

After discussing all possible options an informed consent was obtained and the patient was scheduled for surgery. All necessary vaccinations were performed prior to the operation. She was informed of the possibility of ex vivo surgery and, in case of failure, total splenectomy. The patient was placed in a supine position and the table was slightly titled to the right. A 5 mm scope was first inserted to assess the peritoneal cavity. There were no signs of portal hypertension or any contraindications to proceed with the surgery. A 10 mm trocar was placed in the left upper quadrant, at the intersection with the left mammillary line, 3 inches above the transverse umbilical line. Two 8 mm trocars were placed in the right upper quadrant, one 8 mm in the left lateral quadrant and one periumbilical assistant trocar. The patient was then placed in reverse Trendelenburg and the robot was docked, coming from the patient’s head. The third robotic arm was used to retract the stomach to the left. The gastrocolic ligament was divided allowing access to the lesser sac. The left colonic flexure was mobilized using a combination of robotic hook, ultrasonic shears and bipolar coagulation. The splenocolic ligaments were gently detached. The cyst appeared to occupy the central portion of the spleen and it was felt that standard in vivo partial splenectomy would be impossible. Dissection and vascular control of the proximal splenic artery and vein were carefully achieved. A GelPort^®^ (Applied Medical) was installed through a small midline incision below the umbilicus. The splenic artery and vein were clamped with Bulldogs and divided using the robotic scissors, taking care to leave a long proximal stump for the reconstruction. At this point, the spleen was placed in an endobag and retrieved through the GelPort^®^.

**Fig. 2:**
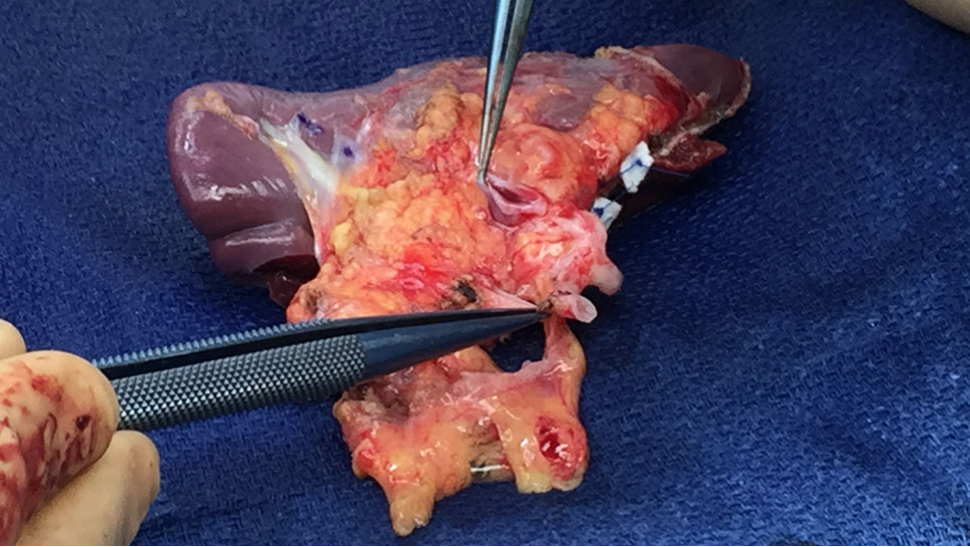
Hemi-spleen after ex vivo bench preparation, ready to be re-introduced into the abdominal cavity

**Fig. 3:**
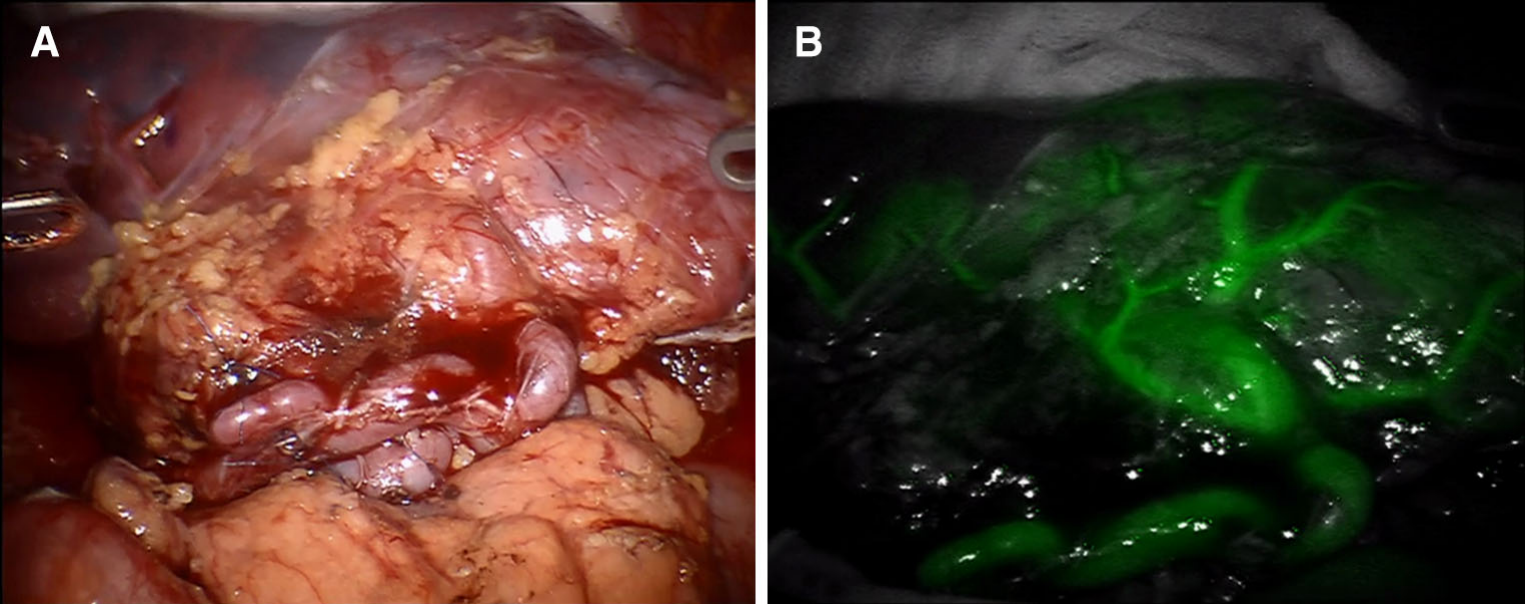
Vascular reconstruction (**a**) and use of ICG that demonstrated optimal perfusion of the transplanted organ (**b**)

The spleen was cooled with infusions at 4 degrees Celsius and flushed with approximately 1.5 L of custodiol solution through the splenic artery until there was a clear flow from the splenic vein. The bench procedure started with the preparation of the hilum. Segmental arteries from the main splenic artery to the upper splenic pole containing most of the cyst were divided. The parenchymal transection was gradually performed until complete resection of the cyst. Hemostasis of the parenchymal transection plane was obtained with multiple prolene 4-0 and 5-0 sutures. Finally, a row of 3/0 interrupted U-stitches with pledgets were placed on the transected capsule (Fig. 2). A leak test with cooled custodiol solution through the splenic artery was performed before re-implantation to ensure good hemostasis after reperfusion. The hemi-spleen was then reintroduced into the abdominal cavity through the midline GelPort^®^. Arterial and venous reconstructions were performed robotically, using running 6-0 Gore-Tex suturing. After vascular reconstruction, indocyanine green fluorescence was used to assess splenic perfusion which was considered to be optimal (Fig. 3). Hemostasis was inspected and was deemed satisfactory after 10 min of observation. The patient was transferred to the surgical intensive care unit (SICU).

## Results

**Fig. 4:**
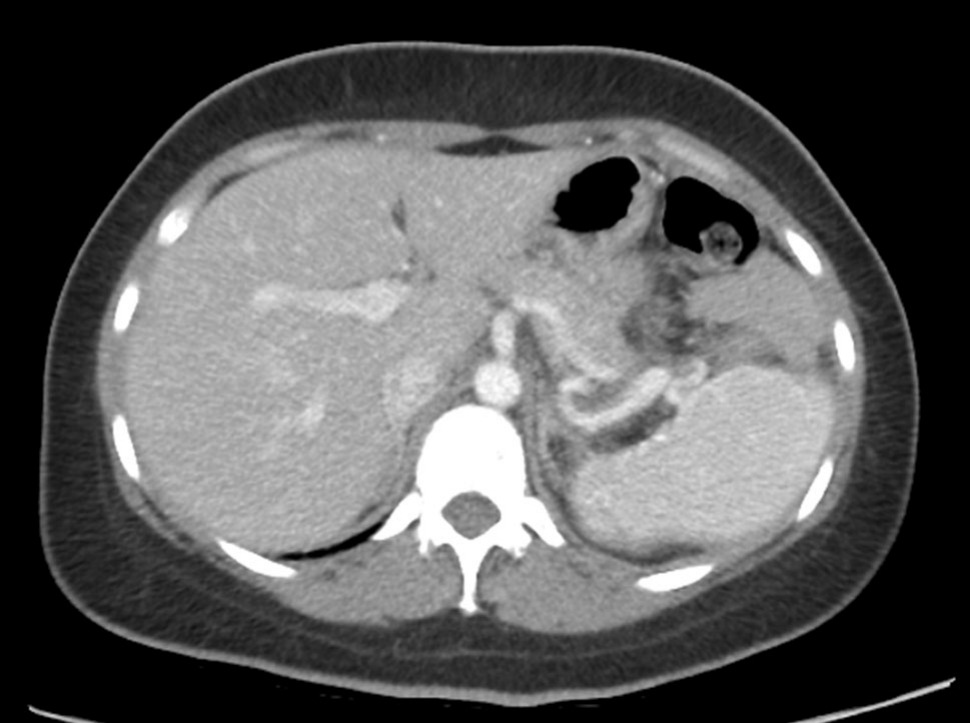
Post-operative CT scan demonstrating patent splenic vessels, homogenously enhanced spleen and no peri-splenic collections

The total operative time was 305 min, with 78 min of robotic time and an estimated blood loss of 150 mL. No intra- or postoperative blood transfusions were necessary. Postoperative ultrasound confirmed a patent arterial and venous flow. The postoperative course was uneventful and the patient was discharged on postoperative day 4. Final pathology report was consistent with benign epithelial splenic cyst. At the 6-month follow-up the patient was doing well. A CT was performed and showed no evidence of perisplenic fluid collection, with a splenic vein that appeared patent and a good size spleen that enhanced homogenously (Fig. 4).

## Discussion

Surgical resection is the mainstay of treatment for larger non parasitic splenic cysts [4]. For many years, and even today, total splenectomy was performed as a treatment for this condition [4]. Unfortunately, the open approach for splenectomy may generate significant morbidity, hence the laparoscopic approach became an option for treatment in the late 1980s and early 1990s [5, 6]. While the minimally invasive approach provides less morbidity and mortality to total splenectomy, the post-operative risk of OPSI is still present, even if minimal [7, 8]. This fact led to the advent of partial splenectomy, which can now be performed robotically and laparoscopically in a safe manner [1, 9]. Unfortunately, large cysts involving the splenic hilum have represented a relative contraindication to minimally invasive partial splenectomy [1].

There have been reports of total splenectomy and splenic tissue re-implantation, especially in cases of trauma [10–12]. While these reports seem interesting, the amount of splenic tissue re-implanted as well as its function level is still under investigation.

The field of organ transplantation has provided new and innovative approaches to resection of tumors initially deemed unresectable. While still investigational, there are now several reports of ex vivo resections of a variety of lesions with re-implantation of the affected organ [13, 14]. Because of the invasive nature of those procedures and the technical challenges, there is to date no large experience of the use of a minimally invasive approach for these cases.

## Conclusions

To our knowledge, there is only one described case of open ex vivo partial splenectomy and re-implantation for non parasitic splenic cyst [2]. We described the first case of minimally invasive, robotic assisted spleen extraction, with ex vivo resection of a large benign splenic cyst and re-implantation of the spleen. We believe this case to be interesting because it proves that partial splenectomy is feasible, even with large cysts involving the splenic hilum. Moreover, it provides evidence that a minimally invasive approach to ex vivo surgery is possible, safe and effective. The robotic technique allowed us to perform a completely intra-abdominal, precise vascular reconstruction of the auto-transplanted organ.

In conclusion, we believe this case provides evidence that the robotic approach may be an asset in ex vivo surgery and that a large splenic cyst involving the splenic hilum is not an absolute contraindication to partial splenectomy.
